# Social Determinants and Developmental Factors Influencing Suicide Risk and Self-Injury in Healthcare Contexts

**DOI:** 10.3390/ijerph22030411

**Published:** 2025-03-11

**Authors:** Marly Johana Bahamón, José Julián Javela, Andrea Ortega-Bechara, Shadye Matar-Khalil, Esteban Ocampo-Flórez, J Isaac Uribe-Alvarado, Andrés Cabezas-Corcione, Lorena Cudris-Torres

**Affiliations:** 1 Facultad de Ciencias de la Salud Montería, Universidad del Sinú “Elías Bechara Zainúm”, Córdoba 230002, Colombia; josejavela@unisinu.edu.co (J.J.J.); a.ortega.bechara@unisinu.edu.co (A.O.-B.); shadyematar@unisinu.edu.co (S.M.-K.);; 2 CINDE, Pontificia Universidad Javeriana, Bogotá 110231, Colombia; eocampo12@gmail.com; 3 Facultad de Psicología, Universidad de Colima, Colima 28040, Mexico; iuribe@ucol.mx; 4 Departamento de Ciencias Sociales, Universidad de la Costa, Barranquilla 080002, Colombia; lcudris3@cuc.edu.co

**Keywords:** social determinants, developmental factors, suicide risk, self-injury, social support, empathy, adolescents

## Abstract

Background: Suicide is a global public health issue, particularly in low- and middle-income countries and among vulnerable groups such as adolescents. Despite increasing research efforts, understanding the psychosocial factors associated with suicidal behavior remains a challenge. This study examines family and personal histories of suicidal behavior, exposure to violence, empathy, and perceived social support in adolescents who have received healthcare services in Ecuador. Methods: A cross-sectional study was conducted with 438 adolescents aged 12 to 18 years. Participants were classified into suicide attempt survivors (AS, *n* = 58) and non-attempters (NAS, *n* = 380). A characterization questionnaire was applied (prior hospitalization for suicide attempt, family history, and survivor condition), the Alexian Brother Urge to Self-Injure scale, the Plutchik Suicide Risk Scale, the Multidimensional Scale of Perceived Social Support, and the Cognitive and Affective Empathy Test. Results: Adolescents with a history of suicide attempts exhibited higher levels of self-injurious behavior impulse (OR = 8.90, CI 95% [4.28–18.52], *p* < 0.001), Gravity attempt (OR = 8.162, CI 95% [4.34–15.37], *p* < 0.001), and suicide risk (OR = 2.90, CI 95% [1.42–5.94], *p* = 0.006). A significant association was found between suicide attempts and exposure to domestic (*p* = 0.000), school (*p* = 0.000), and sexual violence (*p* = 0.000). A family history of suicide attempts increased the likelihood of suicidal behavior in adolescents (OR = 2.40, CI 95% [1.12–5.16], *p* = 0.022). In contrast, perceived family support acted as a potential protective factor (OR = 0.36, CI 95% [0.15–0.91], *p* = 0.055). Conclusions: These findings highlight the need for prevention strategies that address social and developmental factors.

## 1. Introduction

Currently, suicide remains a major global public health concern, particularly in low- and middle-income countries, which account for 77% of cases worldwide. Vulnerable populations, including adolescents, indigenous peoples, refugees, migrants, individuals with diverse sexual orientations and gender identities, and incarcerated individuals, are disproportionately affected [1,2,3,4]. In Ecuador, suicide is the second leading cause of death among children and adolescents aged 10 to 19, with a rate of 7.5 deaths per 100,000 inhabitants, representing 15% to 20% of all deaths due to external causes in this age group. According to reports from the Pan American Health Organization (PAHO), this is the highest rate among neighboring countries, placing Ecuador among the countries with the highest suicide rates in the world [5,6,7,8,9,10,11]. Although research on the evolution of this phenomenon in children and adolescents in the country is limited, the progressive increase in suicide rates over the past two decades is particularly concerning. In this country, research on suicide attempts among children and adolescents is very limited, making it difficult to identify the specific social and psychological factors that contribute to suicidal behavior in this population, thus making targeted prevention efforts more challenging [1,12,13,14,15,16].

This phenomenon has been scarcely explored, although it has been linked to family, emotional, academic, economic, and mental health problems. This situation is particularly concerning for children and adolescents, as it adds to the multiple risk factors that affect their psychosocial development during a stage of great vulnerability, marked by the biological and psychological changes inherent to the transition from childhood to adulthood, which may heighten their vulnerability to suicidal behaviors [6,8,12,17]. The increasing suicide rates among children and adolescents in this country highlight the need for a comprehensive social development strategy that addresses economic, educational, and healthcare disparities. The lack of access to mental health services, combined with the social stigma surrounding suicide, has contributed to the absence of effective interventions [18].

In addition to these elements, numerous risk factors associated with adolescent suicide have been identified, including distal and proximal risk factors at both the intra- and interpersonal levels [12,19,20]. Among the most prevalent factors are adverse childhood events (exposure to violence, physical, sexual, and emotional abuse, bullying, cyberbullying, family instability, and/or abuse), poor academic performance, and substance use [12,21]. Other factors related to adolescent suicide include dysregulated emotional processing, guilt, shame, hopelessness, feelings of loneliness, lack of reasons to live, feelings of worthlessness, poor conflict resolution skills, and coping strategies that contribute to cognitive distortions, in which suicide attempts emerge as a mechanism to escape emotional pain and communicate their distress to others [19,22,23].

Similarly, the broader social environment significantly influences the emotional regulation of adolescents at risk of suicide. Social support systems, including peer relationships, school environments, and access to healthcare services, are key protective factors against suicidal ideation [24]. Studies have shown that adolescents who perceive strong family and social support exhibit lower levels of self-injurious and suicidal behaviors [17]. However, economic disparities, social stigma, and the lack of mental health awareness in certain communities hinder access to these protective resources, further exacerbating the risk of suicidal behavior [25].

Investment in social infrastructure, including integrated community mental health programs and comprehensive school-based interventions, is crucial to mitigating suicide risk among adolescents [26]. Additionally, strengthening family support networks and promoting awareness campaigns can play a fundamental role in suicide prevention, ensuring that vulnerable youth receive timely and appropriate care [27]. Overall, it is estimated that for every suicide death, an average of 25 attempts are reported, highlighting the need to identify and delineate the main suicide risk and protective factors. The impact of a suicide attempt on individuals, especially adolescents, is significant, as they often experience various adverse consequences, showing greater vulnerability to depression, loneliness, and substance dependence [28,29,30].

Compared to adolescents without a history of suicide attempts, those who have attempted suicide are more likely to experience abusive or violent relationships, commit crimes, experience violence, and develop health problems [31,32]. To address the complexity of suicidal behavior in adolescents, it is essential to consider social development frameworks that integrate mental health into broader social policies. Effective suicide prevention strategies should include early childhood interventions, school-based mental health programs, and employment opportunities to alleviate economic stress [26]. Countries that have implemented comprehensive mental health policies and incorporated these programs within educational and social support frameworks have shown a significant reduction in adolescent suicide rates [33].

This underscores the importance of a multidisciplinary approach involving educators, healthcare professionals, and policymakers working together to strengthen social support systems, ensure comprehensive adolescent mental health care, and provide adolescents with the necessary coping resources [34]. Survivors of suicide attempts, i.e., individuals who have previously attempted to take their own lives, also report feelings of shame, prejudice, and stigmatization, as they are often labeled as irresponsible, cowardly, weak, or attention-seeking. This, combined with the internalization of stigma (self-stigma), lowers their self-esteem and increases the risk of another suicide attempt [2,13,35,36,37].

Studies conducted with survivors of suicide attempts have identified several risk factors, such as the presence of interpersonal conflicts (with partners, family members, classmates), childhood trauma, loss of a loved one, romantic breakups, sociocultural stressors, the presence of psychopathologies (behavioral, eating, anxiety, sleep disorders, substance use), as well as socioeconomic factors and limited availability of support services [19,23,38].

Based on the above, this study aims to analyze the social determinants and developmental factors associated with suicide attempts in adolescents receiving healthcare in Ecuador. Specifically, it examines the relationships between suicide attempt history, suicide risk, self-injurious behavior, perceived social support, empathy, and exposure to violence, with the aim of identifying key risks and protective factors associated with suicidal behavior in adolescents.

## 2. Method

### 2.1. Participants

An incidental sample of 438 adolescents aged between 12 and 18 years was obtained, with a mean age of 15.60 years (SD = 1.70). They resided in the province of Tungurahua, one of the regions with the highest suicide rates in Ecuador. These adolescents received care in various healthcare institutions for two months (May and June 2024) for different reasons, including physical conditions and suicide attempts.

The sample was based on suicide survivor status (individuals who had attempted suicide in the last twelve months), identified as the AS group, and a second group not exposed to the survivor condition, identified as NAS. The AS group consisted of 58 cases (M = 16.10; SD = 1.51), of which 26 (44.8%) were male and 32 (55.2%) were female, and the NAS group comprised 380 cases (M = 15.73; SD = 1.77), 190 males (50%) and 190 females (50%).

In accordance with the provisions of the Ministry of Public Health (MPS), which state that both public and private entities can provide care for suicide attempts, public and private healthcare institutions in the city of Ambato were contacted, with priority given to those that handle more complex cases. In total, five public institutions were contacted (including hospitals and health centers) and five private clinics; of these, one public institution and three private institutions agreed to grant access to the target population for the study. For the selection process, contact information was obtained for adolescents who had been receiving treatment for suicide attempts in the past year, as well as contact details for all adolescents who had been treated for other medical conditions during the same period. To be included in the group of adolescent suicide attempt survivors (AS), participants had to have been treated for a suicide attempt, fall within the required age range (12–18 years), and have their legal guardians provide consent for participation upon contact. For the group without a history of suicide attempts (NAS), all adolescents who met the age criteria and had been treated in the same healthcare institutions during the specified period and whose legal guardians consented to their participation in the study were included.

Inclusion criteria were age range, legal permission from parents or legal guardians, voluntarily agreeing to participate, and a level of education appropriate to their age that allowed comprehension of the questions in the instruments. Adolescents who did not have a basic level of academic training to understand the questionnaires were excluded.

### 2.2. Instruments

Characterization Questionnaire: A set of questions regarding age, sex, area of residence, socioeconomic level, and, in cases where the adolescent had been hospitalized for a suicide attempt, the severity of the attempt, family history, and survivor status. To explore the variable of family history of suicide, the questionnaire included inquiries about previous suicide attempts and completed suicides among close relatives, such as parents, grandparents, uncles/aunts, siblings, and cousins. This information was collected using two straightforward and clear questions within the sociodemographic characterization questionnaire, formulated as follows: “Has any member of your family (parents, grandparents, siblings, uncles/aunts, or cousins) attempted suicide?” and “Has any member of your family (parents, grandparents, siblings, uncles/aunts, or cousins) died by suicide?”.

Additionally, this questionnaire included questions about exposure to sexual violence, domestic violence, and school violence based on operational definitions derived from scientific literature. Participants completed a survey-style questionnaire with dichotomous response options (Yes/No) to indicate the presence or absence of violent experiences in their lives. To assess the presence of these types of violence, the following questions were included:

- Sexual violence: “Has anyone ever forced you to engage in or witness acts of a sexual nature against your will?”
- Domestic violence: “Have you witnessed physical aggression between family members within your home?”
- School violence: “Have you been a victim of physical aggression (hitting, pushing, damage to your belongings) at school?”.

The Alexian Brother Urge to Self-Injure ABUSI [39], translated and validated by Bahamón [40], is an instrument that assesses cognitive and emotional aspects of the urge to self-injure in the previous week by evaluating its frequency, intensity, and duration. It is unidimensional and consists of 5 items with 7 response options, scoring from 0 to 6. Internal consistency reported by the authors ranged from α = 0.92 in the initial measurement to α = 0.96 in the second measurement. All items were highly correlated with the overall scale (0.87 to 0.92). The validity indices of this instrument are CFI = 0.998, IFI = 0.998, GFI = 0.995, AGFI = 0.986, RNI = 0.998, NFI = 0.996, RMSEA of 0.033, and SRMR = 0.011. A cutoff point of 6 or more is considered high.

The Plutchik Suicide Risk Scale, validated in Colombia by Suárez-Colorado [41], assesses previous self-harm attempts, current suicidal ideation intensity, feelings of depression, hopelessness, and others. It is scored by giving a value of 1 to all affirmative responses and 0 to negative ones. Scores above 6 indicate suicide risk. Two factors were found in the Colombian adolescent population: suicide risk (items 13, 14, 15) and depressive symptoms (2, 3, 6, 8, 9, 10). Cronbach’s Alpha reliability for depression was 0.72, and for suicide risk was 0.80; McDonald’s Omega was 0.82 and 0.94, respectively. The CFA showed good fit (x2S-B = 26.36, df = 26, *p* = 0.34; NNFI = 1.0, CFI = 1.0, RMSEA = 0.02, 90% CI (0.00–0.05).

The Multidimensional Scale of Perceived Social Support, validated by Trejos, Bahamón, and Alarcón [42], consists of 12 items that evaluate perceived social support through three dimensions: family (3, 4, 8, 11), friends (6, 7, 9, 12), and significant others (1, 2, 5, 10). These 12 items have a 7-point Likert scale ranging from strongly disagree (1) to strongly agree (7). Its internal consistency is 0.84 (95% CI = 0.83–0.86), and CFA indices show a good fit (AGFI = 31,680.98, BIC = 31,824.74, NNFI = 0.946, CFI = 0.975, RMSEA = 0.049).

The Cognitive and affective empathy test TECA [43] consists of 33 questions that assess Empathic Joy, Perspective Taking, Empathic Stress, and Empathic Joy. It has a 5-option Likert scale (1 “Totally Disagree” to 5 “Totally Agree”).

### 2.3. Procedure

Fifteen research assistants underwent a training session covering ethical considerations, questionnaire administration, and handling sensitive topics to ensure consistency and reliability in data collection. The research objective was explained to obtain informed consent from parents or legal guardians, and subsequently, a personal and individual appointment was arranged for the administration of the instruments, which were completed with pen and paper in a neutral location.

Regarding the instruments used, we clarify that all had been previously validated in Latin American contexts, ensuring their suitability for the studied population. However, prior to their application, a pilot test was conducted with 10 adolescents to assess the clarity of the content and ensure their comprehension within the Ecuadorian context. This process confirmed that participants could respond to the items adequately without difficulties in interpretation.

Regarding the sample selection, we have strengthened the description in the Section 2, detailing the inclusion and exclusion criteria used. The process of contacting healthcare institutions has been specified, prioritizing those that handle more complex cases. Additionally, it has been clarified that inclusion in the suicide attempt survivor group (AS) was carried out by verifying their history through self-report and clinical records, while the NAS group was composed of adolescents treated in the same institutions without a history of suicide attempts.

### 2.4. Results Analysis

For this analysis, ages were grouped into preadolescents (12, 13, and 14 years) and adolescents (15, 16, 17, and 18 years) based on developmental stage; the Kolmogorov–Smirnov test was used to assess data normality, identifying that the data did not follow a normal distribution. The Kruskal–Wallis test was applied to compare psychosocial characteristics between the groups of adolescents. Subsequently, to analyze the data, a binary logistic regression analysis was conducted to examine the association between various risk and protective factors and the likelihood of a suicide attempt in adolescents. This statistical technique was chosen as the outcome variable (suicide attempt) is dichotomous. The model estimated logistic regression coefficients (B), standard errors (SEs), odds ratios (ORs), and 95% confidence intervals (CIs) for each predictor variable. The odds ratio (OR) was used to interpret the strength and direction of associations, where values greater than 1 indicate an increased likelihood of the event occurring, while values less than 1 suggest a protective effect. Statistical significance was assessed using *p*-values (<0.050), and multicollinearity diagnostics were performed to ensure the reliability of the model. Data were analyzed using SPSS 25.0 and JASP 19.0 statistical software.

### 2.5. Ethical Considerations

Since the study population consisted of adolescents, some of whom had experienced suicide attempts, strict ethical measures were implemented to ensure their well-being and the protection of their rights throughout the research process. First, the study complied with the ethical principles of the Declaration of Helsinki and was approved by the Ethics Committee of Universidad del Sinú (Act No. 003, approval code UNISINU-003-2024, issued on 30 April 2024). This ensured that the research protocol was reviewed and approved by an independent committee, guaranteeing adherence to the principles of autonomy, beneficence, and justice for the participants.

Additionally, informed consent was obtained from the legal guardians of the adolescents, and assent was secured from the participants themselves, ensuring that they understood the study’s objectives, the voluntary nature of their participation, and their right to withdraw at any time without consequences. Clear and accessible information was provided regarding data confidentiality and the security measures in place. Given that the study addressed sensitive topics such as suicide attempts, self-injury, and exposure to violence, data collection was conducted in a safe and private environment. Specifically, assessments were carried out in a consultation room or office within a higher education institution, where the adolescents attended in a controlled and confidential setting to complete the questionnaires. In cases where participants were unable to travel to the designated location, authorization was requested from their legal guardians to conduct a home visit, ensuring that the instruments were administered in a suitable space within their homes while maintaining privacy and safety for the participant.

The questionnaires were administered by researchers trained in working with vulnerable populations, ensuring that interactions with participants were conducted ethically, respectfully, and without causing additional distress. Furthermore, a safety and referral protocol was established to ensure that any participant exhibiting significant emotional distress or signs of suicide risk was referred to mental health services available at the collaborating institutions. Information about psychological support hotlines and community resources was also provided.

Finally, the confidentiality and anonymity of the collected data were strictly maintained. The data were securely stored and used exclusively for scientific purposes, with all identifying information removed from reports and publications. These considerations ensured that the research was conducted with the highest respect for the rights, dignity, and safety of adolescents, fully adhering to international ethical standards for research involving vulnerable populations.

## 3. Results

The application of the Kolmogorov–Smirnov test identified that the normality assumption was not met (*p* ≤ 0.000); therefore, the Kruskal–Wallis test was applied to compare psychosocial characteristics between the group of adolescent suicide attempt survivors (AS) and non-survivors (NAS). The results showed no significant differences for the variables of gender (*p* = 0.463) and age (*p* = 0.138), while significant differences were identified for the rest of the variables between the two groups: family history of suicide attempt (*p* < 0.001), family history of suicide (*p* < 0.001), exposure to intrafamily violence (*p* < 0.001), exposure to sexual violence (*p* < 0.001), and exposure to school violence (*p* < 0.001). Table 1 shows that the highest percentage of adolescents with suicide attempts are females aged between 15 and 18 (Table 1).

The comparison between protective and suicidal risk factors among AS and NAS groups showed significant differences in self-injurious behavior impulse (*p* < 0.001), suicide risk (*p* < 0.001), family support (*p* = 0.001), and perceived global social support (*p* = 0.028). Table 2 reveals that the impulse towards self-injurious behavior and suicide risk obtained higher scores among adolescent survivors of suicide attempts and that family social support and perceived global social support were lower among adolescents in this group compared to non-survivor adolescents.

Although no significant differences were identified, a higher proportion of adolescent survivors of suicide attempts showed high scores in perspective-taking, emotional understanding, and empathic stress, as well as lower scores in empathic joy. Regarding perceived social support, adolescent survivors of suicide attempts exhibited lower scores in family, friends, and significant others’ social support compared to their peers without a history of suicide attempts.

Table 3 shows that in both age groups, most adolescents who survived a suicide attempt (AS) had a low-severity attempt, with slightly higher proportions of high-severity attempts in the 15–18 age group (6.0%) compared to the 12–14 age group (3.9%). A family history of suicide attempts was found to be more prevalent among older adolescents who attempted suicide (20.5%) compared to younger adolescents (10.8%).

Regarding exposure to violence, adolescents aged 15–18 years who survived a suicide attempt showed slightly higher rates of domestic violence (17.3% vs. 7.8%), sexual violence (6.8% vs. 1.0%), and school violence (14.3% vs. 9.8%) compared to their younger peers. In both age groups, suicide attempt survivors (AS) showed higher levels of impulse toward self-injurious behavior and suicide risk and lower perceived family support compared to their peers who had not attempted suicide (NAS). Specifically, in the 15–18 age group, a higher proportion of adolescents reported high levels of suicide risk (21.1%) and impulse toward self-injurious behavior (20.2%) compared to the 12–14 age group (23.5% and 13.7%, respectively). In contrast, the perceived social support from family, friends, and other significant individuals was lower among AS in both age groups, particularly among older adolescents.

In Table 4, the results of the binary logistic regression analysis indicate that adolescents with a history of suicide attempts are more likely to exhibit risk factors such as impulse toward self-injurious behavior, suicide risk, and severity of previous suicide attempts compared to those without such a history. Specifically, the severity of the prior attempt emerged as the strongest predictor, with an OR = 8.162 (95% CI: 4.34–15.37, *p* < 0.001), indicating that adolescents with more severe prior attempts are eight times more likely to engage in recurrent suicidal behaviors.

Similarly, impulse toward self-harm and suicide risk were highly significant factors, with ORs of 8.904 (95% CI: 4.28–18.52, *p* < 0.001) and 2.903 (95% CI: 1.42–5.94, *p* = 0.006), respectively. These findings suggest that adolescents with a history of suicide attempts are 8.9 times more likely to experience a high impulse toward self-injurious behavior and 2.9 times more likely to exhibit high levels of suicide risk.

Additionally, a family history of suicide attempts was significantly associated with increased risk (OR = 2.404, 95% CI: 1.12–5.16, *p* = 0.022), indicating that these adolescents are 2.4 times more likely to have a family history of suicidal behavior. This finding suggests a potential transgenerational influence on suicidal behavior.

On the other hand, perceived family social support emerged as a significant protective factor in the likelihood of suicide attempts, with an OR of 0.364 (95% CI: 0.15–0.91, *p* = 0.055), suggesting that adolescents with higher levels of family support have a lower risk of attempting suicide. In contrast, perceived social support from friends (OR = 1.253, 95% CI: 0.56–2.82, *p* = 0.646) and other significant individuals (OR = 1.218, 95% CI: 0.55–2.71, *p* = 0.696) did not show a statistically significant relationship with the occurrence of suicide attempts. Similarly, perceived overall social support (OR = 2.268, 95% CI: 0.61–8.46, *p* = 0.238) also did not reach statistical significance, although the direction of the effect suggests a possible relationship with suicide risk.

Additionally, factors such as exposure to domestic, school, and sexual violence showed positive associations with the risk of suicide attempts, although they did not reach statistical significance in this model.

## 4. Discussion

Generally, studies on suicide have overlooked the analysis of suicide attempts, which is problematic because this behavior does not always require medical attention, which limits its registration in healthcare systems [44,45,46,47]. The lack of adequate recording of suicide attempts in healthcare systems is largely due to the absence of social infrastructure that enables timely detection and access to mental health services, especially in low- and middle-income countries [34]. Social determinants such as poverty, lack of access to education, and social discrimination are associated with reduced care access for at-risk adolescents [48]. Strengthening mental health infrastructure and improving data collection can facilitate earlier and more effective interventions [24]. Therefore, this research aimed to analyze the relationship between suicide attempts, risk, and protective factors.

The results highlighted the high number of completed suicides reported in the survey, which explored the suicide histories of participants’ family members and friends. These figures can be explained by psychosocial and cultural issues in Ecuador, including the prevalence of extreme poverty, violence, and family conflicts. These issues make the young population more vulnerable to taking fatal measures due to marked gender inequalities and higher exposure to physical, sexual, and emotional violence [7,9,10,11,49,50].

In the Ecuadorian context, the lack of accessible mental health services and community intervention programs presents a significant challenge in preventing adolescent suicide [26]. Gender inequalities and exposure to violence are exacerbated by the absence of public policies that integrate mental health into social and educational systems [18]. It has been shown that communities with limited access to social and economic support networks have higher rates of suicide attempts, highlighting the importance of strengthening interventions at the community level [25].

Eighteen percent of adolescents surveyed reported a family history of suicide, which places them at higher risk for suicidal behavior themselves, a condition that promotes multiple issues for young people to confront, potentially leading to subsequent suicide attempts or completions. Studies suggest that adolescents affected by family suicides report high levels of thwarted belongingness and depression associated with the circumstances of death, compounded by family distress and feelings of guilt that disrupt their emotional states [16,51,52].

Regarding the prevalence of suicide attempts, this research identified that 13.2% of participants reported a history of suicide attempts, a figure consistent with other studies ranging between 4.1% and 13.2% [53]. These numbers highlight the urgent need for prevention and intervention programs to reduce the likelihood of future suicides among adolescents, considering that a history of suicide attempt is a significant predictor of future attempts and suicide mortality [2,54].

Family social support was identified as a significant protective factor in the prevention of adolescent suicide, whereas perceived social support from friends and other significant individuals did not reach statistical significance. These findings highlight the need for more in-depth analysis, considering that subjective measures were used to assess social support. Furthermore, in contexts with low levels of social and family cohesion, adolescents may face greater barriers to seeking help, underscoring the importance of further investigating the differential role of various sources of support in suicide prevention [17].

Regarding the gender of participants with a history of suicide attempts, recent research with adolescents has shown that females are more likely than males to have attempted suicide and to have suicidal thoughts. Nevertheless, contrary to previous studies, this research found a similar proportion of women and men with a history of suicide attempts (14.3% in women and 12% in men). However, some studies found results similar to those found in the present study [3,55]. Two countries, Ecuador and Mexico, are currently experiencing very similar violence issues [56,57,58]. Additionally, no significant differences were found for age groups, although older adolescents (15–18 years) exhibited higher rates of this behavior, partially coinciding with previous studies reporting a higher number of attempts among adolescents aged 13 to 19 years [53].

Regarding exposure to different types of violence, the findings indicate a significant difference between adolescents who have attempted suicide and those who have not, and it is related to exposure to various types of violence. This research showed that adolescents exposed to intrafamily violence, sexual violence, and school violence were more likely to attempt suicide, consistent with other studies suggesting that a high frequency of victimization increases the odds of suicide attempts in adolescents by 12 times [59,60]. Other authors have also identified that one in five adolescents who have experienced at least one form of violence has attempted suicide [61,62,63].

Gender differences in suicide attempts reflect social and cultural patterns that influence risk perception and help-seeking behavior [64]. Although a higher prevalence of suicide attempts has traditionally been identified in women, the normalization of risky behaviors in men may lead to an underestimation of the problem in this population [27] It is crucial to adopt gender-sensitive prevention approaches that consider differences in adolescent socialization and the specific risk factors for each group [34].

Results regarding self-injurious behavior and suicide risk in adolescents showed significant differences between adolescents with and without a history of suicide attempts, with higher scores for these variables among participants who had attempted to take their own lives. These results suggest that self-injurious behavior is a significant predictor of suicide attempts, as well as an element that increases suicide risk, which should be considered in the comprehensive approach to suicidal behavior [49,65,66].

While the findings of this study provide significant evidence regarding risk and protective factors associated with suicide attempts in adolescents, their generalizability at the national level should be interpreted with caution due to the sample’s focus on the Tungurahua province. This region has one of the highest adolescent suicide rates in Ecuador, allowing for an examination of these dynamics in a high-vulnerability context. However, given that Ecuador is a country with sociocultural diversity and economic inequalities, the expression of suicide risk may vary based on factors such as access to mental health services, levels of violence, social support networks, and differences in the stigmatization of suicide across communities.

Despite the relevance of the findings, the study presents some methodological limitations that should be considered when interpreting the results. One of the main limitations lies in the imbalance between the number of adolescents with a history of suicide attempts (SA) and those without such a history (NSA), as the proportion of NSA adolescents was higher. Although this phenomenon is expected due to the lower prevalence of suicide attempts in the general population, the difference in sample sizes may affect statistical power and the generalizability of the findings. However, this limitation was mitigated through the use of binary logistic regression analysis, a statistical technique that allows for the evaluation of multiple factors’ effects on the odds ratio (OR) of suicide attempts, regardless of the sample imbalance. This approach enabled the identification of the magnitude of associations between independent variables and suicide attempts.

Another important limitation is that the study employed a cross-sectional design, which prevents the establishment of causal relationships between the variables assessed. While significant associations between risk factors and suicide attempts were identified, it is not possible to determine whether exposure to adverse events preceded suicidal behavior or if both phenomena interact in a dynamic process. Additionally, the use of self-report instruments to assess risk and protective factors may have influenced the accuracy of responses, as adolescents might underestimate or overestimate certain events due to memory biases, social desirability, or difficulties in recognizing traumatic experiences, such as domestic violence or sexual abuse.

Furthermore, although family history of suicidal behavior was included, the information obtained was based on adolescents’ perceptions of these events rather than clinical records or other external sources that could validate these reports.

These results have important implications in intervention processes for adolescents, as having high scores in perspective-taking, emotional understanding, and empathic stress allows arguments related to their connection with significant others to be used to dissuade adolescents from suicidal behavior as an option in the future, reducing suicide risk, unlike what happens with adults [18].

The implementation of intervention programs aimed at strengthening adolescents’ resilience and social development has proven to be an effective strategy for reducing suicide risk [26]. School-based suicide prevention programs, along with emotional education, have been shown to improve the perception of social support and reduce risk factors associated with self-injury [25]. Integrating these programs into a broader public health strategy can significantly contribute to reducing suicide attempt rates in vulnerable populations.

Regarding the social support variable, the data revealed significant differences between the group with a history of suicide attempts and the group of adolescents without such a history, with lower scores in the former group. This finding aligns with previous studies that have identified negative correlations between social support, suicidal ideation, and suicide attempts in adolescent populations. However, when analyzing different types of social support, only family support showed a statistically significant association with a lower likelihood of suicide attempts, whereas perceived support from friends and other significant individuals did not reach statistical significance. Nevertheless, studies conducted by McClay and other researchers have found that social support, in general, may play a role in the disclosure of suicide attempts, facilitating access to help and reducing barriers to intervention [1,48,67].

In this regard, the only protective factor that showed statistical significance in this study was family social support, suggesting that having a support network within the family unit may reduce the risk of suicide attempts in adolescents. Although other studies have indicated that social support, in general, may play a protective role in populations such as homeless adolescents, opioid users, and adolescents in general, this analysis did not find statistically significant evidence for support from friends and other significant individuals, this analysis did not find statistically significant evidence to support this effect for social support from friends and other significant individuals. These results have implications for the design of prevention processes, emphasizing the importance of strengthening family support as a key element in reducing risk behaviors while also highlighting the need to continue exploring the role of other sources of support in future studies [68,69,70].

Overall, the results of this study point to family history of suicidal behavior, exposure to different types of violence, and deficits in empathy as risk factors among adolescents for suicide attempts, highlighting the need for intervention processes in this population considering they are at higher risk than the rest of the population for suicide attempts and possibly for completed suicide [49,66].

These results should be interpreted with caution, given that this was a cross-sectional study, and the causality of the attempt may involve more aspects. Therefore, it will be necessary in future research to analyze other elements such as the severity of the attempt, the temporality of the attempt, and the number of attempts made by adolescents. Additionally, establishing causal relationships between empathy and suicide attempts will be necessary to determine if it can be a cause of behavior or an effect of stigma due to the execution of the behavior. Another aspect to consider will be addressing adolescents’ exposure to violence and examining different sources of support, as it will be necessary to establish which sources are most effective according to the characteristics of young people.

The findings of this research can contribute to the development of public policies aimed at preventing adolescent suicide by strengthening family and community support networks. It is essential that governmental strategies include training programs for families and community organizations, equipping them with tools for the early recognition of risk signs and the proper initial response to suicidal behavior. Additionally, the integration of multidisciplinary teams in healthcare centers and schools should be prioritized, ensuring they are trained to identify and refer at-risk adolescents. Policies should focus on training teachers and community leaders in psychological first aid and emotional support to ensure timely assistance for adolescents who receive timely assistance before their situation worsens. Finally, it is crucial to promote awareness campaigns and reduce the stigma associated with seeking help, facilitating access to mental health services within educational and community settings.

## 5. Conclusions

The findings of this study reveal significant differences between certain risk and protective factors among adolescents with and without a history of suicide attempts. Descriptive results suggest significant differences between these groups regarding the history of suicidal behavior within the family and exposure to domestic, sexual, and school violence. Furthermore, the logistic regression analysis identified that the severity of prior suicide attempts was the strongest predictor of recurrent attempts, with a likelihood eightfold higher among adolescents with more severe prior attempts. Additionally, self-harm impulse and suicide risk were significantly associated with previous suicide attempts, notably increasing the probability of recurrence.

Regarding protective factors, family social support was found to reduce the likelihood of suicide attempts. Although perceived support from friends and other important people did not reach statistical significance in the regression model, the data suggest a potential protective effect against suicidal behavior in adolescents.

## Figures and Tables

**Table 1:** Frequencies of psychosocial characteristics and suicide attempt.

Psychosocial Characteristics	Suicide Attempt Survivors NAS (No, *n* = 380)		Suicide Attempt Survivors AS (Yes, *n* = 58)		Total (N = 438)	
	*n*	(%)	N	(%)	*n*	(%)
Age						
12–14 years	93	(91.2)	9	(8.8)	102	(23.1)
15–18 years	287	(85.5)	49	(14.5)	336	(76.8)
Sex						
Women	190	(85.7)	32	(14.3)	222	(50.6)
Men	190	(88.0)	26	(12.0)	216	(49.4)

**Table 2:** Frequencies of risk factors, protective factors, and suicide attempts.

Psychosocial Characteristics	Suicide Attempt Survivors NAS (No, *n* = 380)		Suicide Attempt Survivors AS (Yes, *n* = 58)		Total (N = 438)	
	*n*	(%)	N	(%)	*n*	(%)
Gravity attempt						
Low	--	(---)	43	(58.6)	---	(---)
High	--	(---)	15	(41.3)	---	(---)
Family history of suicide attempt						
No	322	(90.3)	35	(9.7)	357	(81.6)
Yes	58	(71.6)	23	(28.4)	81	(18.4)
Family history of suicide						
No	320	(89.2)	39	(10.8)	359	(82.0)
Yes	60	(75.9)	19	(24.1)	79	(18.0)
Victim of domestic violence						
No	331	(89.3)	40	(10.7)	371	(84.7)
Yes	49	(73.1)	18	(26.9)	67	(15.2)
Victim of sexual violence						
No	363	(87.7)	51	(12.3)	414	(94.5)
Yes	17	(70.8)	7	(29.2)	24	(5.4)
Victim of school violence						
No	335	(88.2)	45	(77.6)	380	(86.8)
Yes	45	(11.8)	13	(22.4)	58	(13.2)
Perspective adoption						
Low	171	(87.8)	24	(12.2)	196	(44.7)
High	208	(86.0)	34	(14.0)	242	(55.2)
Emotional understanding						
Low	138	(87.4)	20	(12.6)	158	(31.6)
High	242	(86.5)	38	(13.5)	280	(63.8)
Empathic stress						
Low	181	(88.0)	25	(12.0)	207	(47.2)
High	198	(85.8)	33	(14.2)	231	(52.7)
Joy empathic						
Low	160	(85.2)	28	(14.8)	188	(42.9)
High	220	(88.0)	30	(12.0)	250	(57.1)
Impulse to self-injurious behavior						
Low	328	(92.2)	28	(7.8)	356	(81.1)
High	52	(63.9)	30	(36.1)	82	(18.8)
Suicide risk						
Low	308	(95.7)	14	(4.3)	322	(73.4)
High	72	(62.4)	44	(37.6)	116	(26.5)
Family social support						
Low	170	(80.7)	41	(19.3)	211	(48.1)
High	210	(92.5)	17	(7.5)	227	(51.8)
Social support friends						
Low	207	(86.3)	33	(13.7)	240	(54.7)
High	173	(87.4)	25	(12.6)	198	(45.2)
Social support others						
Low	170	(84.7)	31	(15.3)	201	(45.9)
High	209	(88.6)	27	(11.4)	236	(53.8)
Overall social support						
Low	182	(84.7)	33	(15.3)	215	(49.0)
High	198	(88.8)	25	(11.2)	223	(50.9)

**Table 3:** Frequencies of risk factors, protective factors, and suicide attempts by age range.

Age Range	Psychosocial Characteristics	Suicide Attempt Survivors NAS (No, *n* = 380)		Suicide Attempt Survivors AS (Yes, *n* = 58)	
		*n*	(%)	N	(%)
	Gravity attempt				
12–14	Low	0	(0.0)	98	(96.1)
	High	0	(0.0)	4	(3.9)
15–18	Low	0	(0.0)	316	(94.0)
	High	0	(0.0)	20	(6.0)
12–14	Family history of suicide attempt				
	No	83	(89.2)	91	(89.2)
	Yes	10	(10.8)	11	(10.8)
15–18	No	239	(83.2)	267	(79.5)
	Yes	48	(16.7)	69	(20.5)
12–14	Family history of suicide				
	No	85	(91.3)	88	(80.6)
	Yes	8	(8.6)	14	(19.3)
15–18	No	235	(81.8)	271	(81.0)
	Yes	52	(18.1)	65	(19.0)
12–14	Victim of domestic violence				
	No	88	(94.6)	94	(92.2)
	Yes	5	(5.4)	8	(7.8)
15–18	No	243	(84.7)	278	(82.7)
	Yes	44	(15.3)	58	(17.3)
12–14	Victim of sexual violence				
	No	93	(100)	101	(99.0)
	Yes	0	(0)	1	(1.0)
15–18	No	270	(94.0)	313	(93.2)
	Yes	17	(6.0)	23	(6.8)
12–14	Victim of school violence				
	No	85	(91.3)	92	(90.2)
	Yes	8	(8.7)	10	(9.8)
15–18	No	250	(87.0)	288	(85.7)
	Yes	37	(13.0)	48	(14.3)
12–14	Perspective adoption				
	Low	36	(38.7)	38	(37.3)
	High	57	(61.3)	64	(62.7)
15–18	Low	137	(47.7)	158	(47.0)
	High	150	(52.3)	178	(53.0)
12–14	Emotional understanding				
	Low	30	(32.3)	34	(33.3)
	High	63	(67.7)	68	(66.7)
15–18	Low	107	(37.3)	124	(37.0)
	High	180	(62.7)	212	(64.0)
12–14	Empathic stress				
	Low	49	(52.7)	50	(51.0)
	High	44	(47.3)	50	(49.0)
15–18	Low	132	(46.0)	154	(45.8)
	High	155	(54.0)	182	(54.2)
12–14	Joy empathic				
	Low	32	(34.4)	36	(35.3)
	High	61	(65.6)	66	(64.7)
15–18	Low	128	(44.6)	152	(45.2)
	High	159	(55.4)	184	(54.8)
12–14	Impulse to self-injurious behavior				
	Low	83	(89.2)	88	(86.3)
	High	10	(10.7)	14	(13.7)
15–18	Low	244	(85.0)	268	(79.3)
	High	43	(15.0)	68	(20.2)
12–14	Suicide risk				
	Low	76	(81.7)	78	(76.5)
	High	17	(18.3)	24	(23.5)
15–18	Low	231	(80.5)	245	(72.9)
	High	56	(19.5)	91	(21.1)
12–14	Family social support				
	Low	35	(37.6)	40	(39.2)
	High	58	(62.4)	62	(61.0)
15–18	Low	136	(47.4)	171	(50.9)
	High	151	(52.6)	165	(49.1)
12–14	Social support friends				
	Low	47	(50.5)	53	(52.0)
	High	46	(49.4)	49	(48.0)
15–18	Low	159	(55.4)	188	(56.0)
	High	128	(44.6)	148	(44.0)
12–14	Social support others				
	Low	39	(41.9)	44	(43.1)
	High	54	(58.1)	58	(56.9)
15–18	Low	132	(46.0)	159	(47.3)
	High	155	(54.0)	177	(52.7)
12–14	Overall social support				
	Low	40	(43.0)	45	(44.1)
	High	53	(57.0)	57	(55.9)
15–18	Low	142	(49.5)	171	(50.9)
	High	145	(50.5)	165	(49.1)

**Table 4:** Results of the binary logistic regression analysis for risk and protective factors in adolescents with suicide attempt.

Predictor Variable	B	SE	OR	95% CI		*p*-Value
				Lower	Upper	
Constant	−2.581	0.208	0.076	0.00	0.00	<0.001 **
Gravity attempt	2.099	0.521	8.162	4.34	15.37	<0.001 **
Family history of suicide attempt	0.877	0.382	2.404	1.12	5.16	0.022 *
Family history of suicide	0.255	0.386	1.290	0.67	2.48	0.510
Victim of domestic violence	0.395	0.439	1.484	0.69	2.48	0.368
Victim of sexual violence	0.210	0.645	1.234	0.35	4.34	0.745
Victim of school violence	0.423	0.461	1.527	0.64	3.64	0.358
Perspective adoption	0.148	0.418	1.160	0.51	2.64	0.723
Emotional understanding	0.215	0.421	1.240	0.53	2.87	0.609
Empathic stress	0.139	0.350	1.149	0.58	2.28	0.692
Joy empathic	0.040	0.403	1.041	0.50	2.16	0.921
Impulse to self-injurious behavior	2.187	0.389	8.904	4.28	18.52	<0.001 **
Suicide risk	1.066	0.366	2.903	1.42	5.94	0.006 *
Family social support	−1.010	0.525	0.364	0.15	0.91	0.055
Social support friends	0.525	0.490	1.253	0.56	2.82	0.646
Social support others	0.250	0.505	1.218	0.55	2.71	0.696
Overall social support	0.819	0.694	2.268	0.61	8.46	0.238

Note: B = Logistic regression coefficient; SE = Standard Error; OR = Odds Ratio; 95% CI (Lower–Upper) = 95% Confidence Interval. An OR > 1 indicates an increased likelihood of the event occurring, whereas an OR < 1 suggests a protective effect; *p*-values < 0.050 are considered statistically significant. Multicollinearity was assessed, and no major issues were detected. * values with statistical significance (*p* < 0.05); ** values with high statistical significance (*p* < 0.001).

## Data Availability

Upon request, the data used in the current study are available through Dr. Marly Johana Bahamón (email: marlybahamon@unisinu.edu.co) and will be provided on a case-by-case basis.
